# Effect of Angiotensin(1-7) on Heart Function in an Experimental Rat Model of Obesity

**DOI:** 10.3389/fphys.2015.00392

**Published:** 2015-12-21

**Authors:** Katja Blanke, Franziska Schlegel, Walter Raasch, Michael Bader, Ingo Dähnert, Stefan Dhein, Aida Salameh

**Affiliations:** ^1^Department of Pediatric Cardiology, Heart Center Leipzig, University of LeipzigLeipzig, Germany; ^2^Clinic for Cardiac Surgery, Heart Center Leipzig, University of LeipzigLeipzig, Germany; ^3^Institute of Experimental and Clinical Pharmacology and Toxicology, University of LübeckLübeck, Germany; ^4^Max-Delbrück Center for Molecular Medicine, Helmholtz-GemeinschaftBerlin, Germany

**Keywords:** angiotensin(1-7), cafeteria diet, heart, obesity, transgenic rats

## Abstract

**Aim:** Obesity is a risk factor for the development of cardiovascular diseases. Recently it was shown that overexpression of the Mas-receptor antagonist angiotensin(1-7) could prevent from diet-induced obesity. However, it remained unclear whether diet-induced obesity and angiotensin(1-7) overexpression might also have effects on the cardiovascular system in these rats.

**Methods:**Twenty three male Sprague Dawley rats were fed with standard chow (SD+chow, *n* = 5) or a cafeteria diet (SD+CD, *n* = 6) for 5 months. To investigate the effect of angiotensin(1-7) transgenic rats, expressing an angiotensin(1-7)-producing fusion protein in testis were used. These transgenic rats also received a 5 month's feeding period with either chow (TGR+chow, *n* = 6) or cafeteria diet (TGR+CD, *n* = 6), respectively. Hemodynamic measurements (pressure-volume loops) were carried out to assess cardiac function and blood pressure. Subsequently, hearts were explanted and investigated according to the Langendorff technique. Furthermore, cardiac remodeling in these animals was investigated histologically.

**Results:**After 5 months cafeteria diet feeding rats showed a significantly increased body weight, which could be prevented in transgenic rats. However, there was no effect on cardiac performance after cafeteria diet in non-transgenic and transgenic rats. Moreover, overexpression of angiotensin(1-7) deteriorated cardiac contractility as indicated by impaired dp/dt. Furthermore, histological analysis revealed that cafeteria diet led to myocardial fibrosis in both, control and transgenic rats and this was not inhibited by an overproduction of angiotensin(1-7).

**Conclusion:**These results indicate that an overexpression of circulating angiotensin(1-7) prevents a cafeteria diet-induced increase in body weight, but does not affect cardiac performance in this experimental rat model of obesity. Furthermore, overexpression of angiotensin(1-7) alone resulted in an impairment of cardiac function.

## Introduction

According to the World Health Organization obesity is one of the leading risks for global deaths. Since the mid-Twentieth century, the prevalence of obesity has increased substantially and its importance as a main risk factor for the development of metabolic disorders like diabetes and metabolic syndrome, and cardiovascular diseases has been rapidly evolving. One of the major cause of overweight and obesity is unhealthy diet. Several animal models of obesity have been described, including diet-induced obesity in rats, which closely reflects the situation of human obesity (Lutz and Woods, 2012).

In their experimental study Müller-Fielitz et al. demonstrated that 19 weeks of high-caloric diet in rats resulted in metabolic syndrome, characterized by obesity, insulin resistance, hyperphagia, hyperlipidaemia and hypertension (Müller-Fielitz et al., 2015).

The pathophysiology of obesity and its associated cardiometabolic alterations are complex and further investigations are needed to clarify the underlying molecular mechanisms. However, there is evidence that an association exists between the renin-angiotensin-aldosterone system (RAAS) and a number of cardiac and metabolic disorders (Santos et al., 2005, 2008, 2010, 2012; Müller-Fielitz et al., 2015).

In the past years, our understanding of the RAAS has dramatically changed, since evidence occurred revealing the existence of a novel arm of this system, the ACE2/angiotensin peptide 1-7 (Ang(1-7))/Mas receptor axis (Santos et al., 2013a,b).

ACE2 is a transmembrane peptide, converting AngI into the octapeptide Ang(1-9) and AngII into the heptapeptide Ang(1-7). As previously described, the newly identified ACE2/Ang(1-7)/Mas receptor axis seems to be a physiological antagonist of the conventional ACE/AngII/AT_1_ receptor arm (Patel et al., 2014) having vasodilative, antiarrhythmic, antifibrotic, antithrombotic, antihypertrophic, antiangiogenic and antiproliferative properties (Passos-Silva et al., 2013; Santos, 2014).

In accordance with these findings, it would be conceivable that ACE2 and Ang(1-7) may also be involved in diet-induced obesity. In this regard, Gupte and colleagues observed an increase in ACE2 concentration in adipose tissue of mice, fed with high-fat diet (Gupte et al., 2008). Very recently, it could be shown by members of our group that Ang(1-7) overexpression in rats could antagonize the diet-induced obesity (Schuchard et al., 2015). However, it remained unclear, whether these positive effects are also detectable regarding the cardiovascular performance.

The initial idea of our study was, that CD may lead to a cardiac phenotype similar to hypertensive heart disease with increased tissue fibrosis and that Ang(1-7) overexpression might antagonize these effects. Since cardiac fibrosis very early leads to prolongation of the activation process with prolonged QRS interval in the edpicardial mapping (Dhein and Hammerath, 2001) we included *in vitro* Langendorff experiments on isolated hearts with extracellular multi-electrode mapping.

## Materials and methods

The following experiments were approved by the Animal Welfare Committee of the city of Leipzig and have been performed according to the Guide for the Care and Use of Laboratory Animals (US National Institute of Health (publication No. 85–23, revised 1996). Body parameters as well as clinical parameters were determined under non-blinded conditions. In contrast, for epicardial mapping and histological analysis investigator was blinded.

### Animal model

All animals of our study were previously included into another study. In this study the working group of Prof. Dr. Raasch, Institute of Experimental and Clinical Pharmacology and Toxicology of the University of Lübeck investigated the effect of cafeteria diet (CD) and an overproduction of Ang(1-7) on metabolic parameters like body weight, food intake and insulin sensitivity at day 147 or 154 as described in the manuscript of Schuchard et al. (2015). For measurement of the insulin sensitivity an insulin tolerance test was carried out: 0.6 international units per kg body weight were administered subcutaneously and glucose levels were determined in blood samples during a 360 min period. Total energy intake was also assessed.

After finishing their measurements animals were delivered on day 160 to the heart center for cardiac investigation.

Thirty four weeks old male Sprague Dawley (SD) rats (Saint Berthevin, Cedex, France) (*n* = 23), obtained from Prof. Dr. Raasch, were investigated. Male transgenic rats (TGR, *n* = 12), overexpressing Ang(1-7) were generated by Prof. Dr. Michael Bader (Max-Delbrück-Center for Molecular Medicine in Berlin). In this transgenic rat model, an Ang(1-7)-producing fusion protein (TGR(A1-7)3292) was expressed in the testis of the rats. This fusion protein is then cleaved in the testis tissue resulting in an about 4.5-fold increase in testicular Ang(1-7) and a 2.5-fold increase in circulating Ang(1-7) (Schuchard et al., 2015). In detail, the generation of these transgenic rats was described previously by Santos et al. (2004).

A total of 23 rats were maintained in pairs and under controlled conditions at room temperature and with a 12:12 h light/dark cycle. Water was provided *ad libitum*.

### Feeding

At 12 weeks of age rats were divided into four groups depending on their genetic background and the 5 month's feeding procedure. The first group were wild type (WT) rats only fed on standard laboratory diet (SD+chow, *n* = 5). The second group included WT rats, which had free access to standard chow and cafeteria diet (CD) (SD+CD, *n* = 6). As previously described, CD consisted of commercial chocolate and cookies (62% carbohydrates, 25% fat, 6% protein, 2% fiber). Rats received only one kind of sweet per day (Müller-Fielitz et al., 2015; Schuchard et al., 2015). Furthermore, chocolate and cookie bars were changed daily in a regular manner (Müller-Fielitz et al., 2015; Schuchard et al., 2015). The third group were transgenic (TGR) rats, receiving standard chow food (TGR+chow, *n* = 6). The transgenic rats of the fourth group could also freely choose between chow and CD (TGR+CD, *n* = 6).

### Study protocol

After 5 month of feeding rats were weighted (once per rat) and submitted to the final experiment. Therefore, the rats were anesthetized by intramuscular injection with a mixture of fentanyl (0.005 mg/kg body weight), midazolam (2 mg/kg body weight) and medetomidin (0.15 mg/kg body weight). Afterwards, they were intubated and anesthesia was maintained using isoflurane (0.8–1%). To avoid blood clotting heparin (1000 E/kg body weight) was injected into the tail vein.

### Echocardiography

After rats were anesthetized transthoracic echocardiography was performed using a GE Vivid I ultrasound system (ultrasonic probe 3S, GE Healthcare, Munich, Germany). The left ventricle was examined in the parasternal short axis view and fractional shortening as well as ejection fraction were calculated to assess left ventricular function. A total of five measurements per rat were carried out to obtain mean EF and FS, respectively.

### Pressure-volume-loops

For hemodynamic measurements a 4-segment Millar catheter (SPR 869, Millar instruments, Houston, USA) was inserted into the left ventricle of the anesthetized rats via the left carotid artery. The pressure-volume-relationship of the left ventricle was recorded under baseline conditions and during stimulation of the beta-adrenoceptor by dobutamine (single dose of 0.2 mg/kg body weight). The following hemodynamic parameters were measured using a signal amplifier (MPVS Ultra, Millar instruments, Houston, USA) and for data acquisition the Power Lab and Lab Chart Software (Föhr Medical Instruments, Seeheim, Germany) was used: heart rate, maximal enddiastolic (LVEDP) and endsystolic (LVESP) left ventricular pressure, maximal systolic contraction velocity (dp/dt(max)) and maximal diastolic relaxation velocity (dp/dt(min)). To determine arterial blood pressure the maximum (systolic) and minimum (diastolic) pressure in the left carotid artery was measured under baseline conditions using the Millar catheter before inserting the catheter into the chamber of the left ventricle. A total of five pressure-volume-loops per rat were recorded to assess hemodynamic parameters.

After completion of hemodynamic recordings the thorax was opened by thoracotomy and hearts were removed for further experiments. Subsequently, the weight of the hearts was determined (once per rat).

### Epicardial mapping

The excised rat hearts were prepared according to the Langendorff-technique as described in detail previously by Dhein et al. (1993).

At a constant pressure of 75 cmH_2_O and a temperature of 37°C hearts were perfused with Tyrode's solution (Na^+^ 161.02 mmol L^−1^, K^+^ 5.36 mmol L^−1^, Ca^2+^ 1.8 mmol L^−1^, Mg^2+^ 1.05 mmol L^−1^, Cl^−^ 147.86 mmol L^−1^, ${\text{HCO}}_{3}^{-}$ 23.8 mmol L^−1^, ${\text{PO}}_{4}^{2-}$, and glucose 11.1 mmol L^−1^; pH 7.4), equilibrated with 95% O_2_ and 5% CO_2_. To measure the left ventricular pressure (LVP) a balloon was fit into the left ventricle via the left atrium. Furthermore, the coronary flow (CF g^−1^ heart weight) and the basic cycle length (BCL) were monitored. To evaluate the electrical excitation epicardial potential mapping was performed according to Dhein et al. (1993). A total of 256 AgCl electrodes in four polyester plates (each 8 × 8 electrodes with an inter-electrode distance of 1 mm) were attached to the surface of the rat hearts in an elastic manner, allowing the electrodes to follow the movement of the heart. Using a computer-assisted 256-channel mapping system (HAL4, Ing. Büro P. Rutten, Hamburg, Germany) 256 unipolar extracellular potentials were recorded simultaneously. According to Durrer and van der Tweel the activation time points at each electrode were defined as the time point of the most negative intrinsic deflection (t(dU/dtmin)) (Durrer and van der Tweel, 1954). Moreover, peak to peak amplitude of the extracellular potentials (PTP), PQ and QRS duration, as well as total activation time (TAT) were assessed. TAT was defined as the time between the activation of the first and the last electrode in a certain heart region. Epicardial mapping experiments were performed once per rat.

### Histological analysis

For histological analysis hearts were fixed in 4% buffered formaldehyde solution for 1 week and stored in 70% ethanol until further processing. Afterwards, hearts were embedded in paraffin, 2 μm thick sections were cut and prepared for histological analysis. Investigations of the histological stained slides were performed using a Zeiss Axiolab microscope and the software AxioVision 4.6 (Zeiss, Jena, Germany).

Conventional hematoxylin-eosin-staining (HE) were carried out to determine the capillary to muscle fiber ratio. 10 visual fields of fasciated myocardial cells per rat were acquired at × 400 magnification and analyzed using the image processing program ImageJ 1.47 (Java) and the ratio of capillary to muscle fiber was determined. Furthermore, 2 μm sections of the paraffin embedded rat hearts were stained with Picrosirius red to evaluate cardiac fibrosis. Using the image analysis program SigmaScan Pro 5 (Jandel Scientific, Erkrath, Germany) 10 visual fields per rat were photographed at × 200 magnification and assessed concerning the percentage of total collagen content within the myocardium.

For histological analysis a total of 50 (SD+chow) and 60 (SD+CD; TGR+chow; TGR+CD), respectively, visual fields of myocardium were investigated.

### Measurement of the femur length

To further examine the influence of CD feeding and overexpression of Ang(1-7) on body parameters of the rats, the femur was prepared and the length was measured (once per rat).

### Statistical analysis

All data are expressed as means ± SEM of *n* experiments. Statistical analyses were performed using Analysis of Variance (ANOVA), if the assumption of normal distribution and equal variance between the groups are met. If ANOVA indicated significance subsequently pairwise comparison by Tukey's *post-hoc* test was done. In case of non-normal distribution and/or the variance was not similar between the groups Kruskal-Wallis test was carried out followed by pairwise comparison using the Dwass-Steele-Chritchlow-Fligner. Significance has been accepted at a significance level of *p* < 0.05. Analyses were performed using Systat 11.0 for Windows software (Jandel Scientific, Erkrath, Germany).

## Results

**Figure 1:**
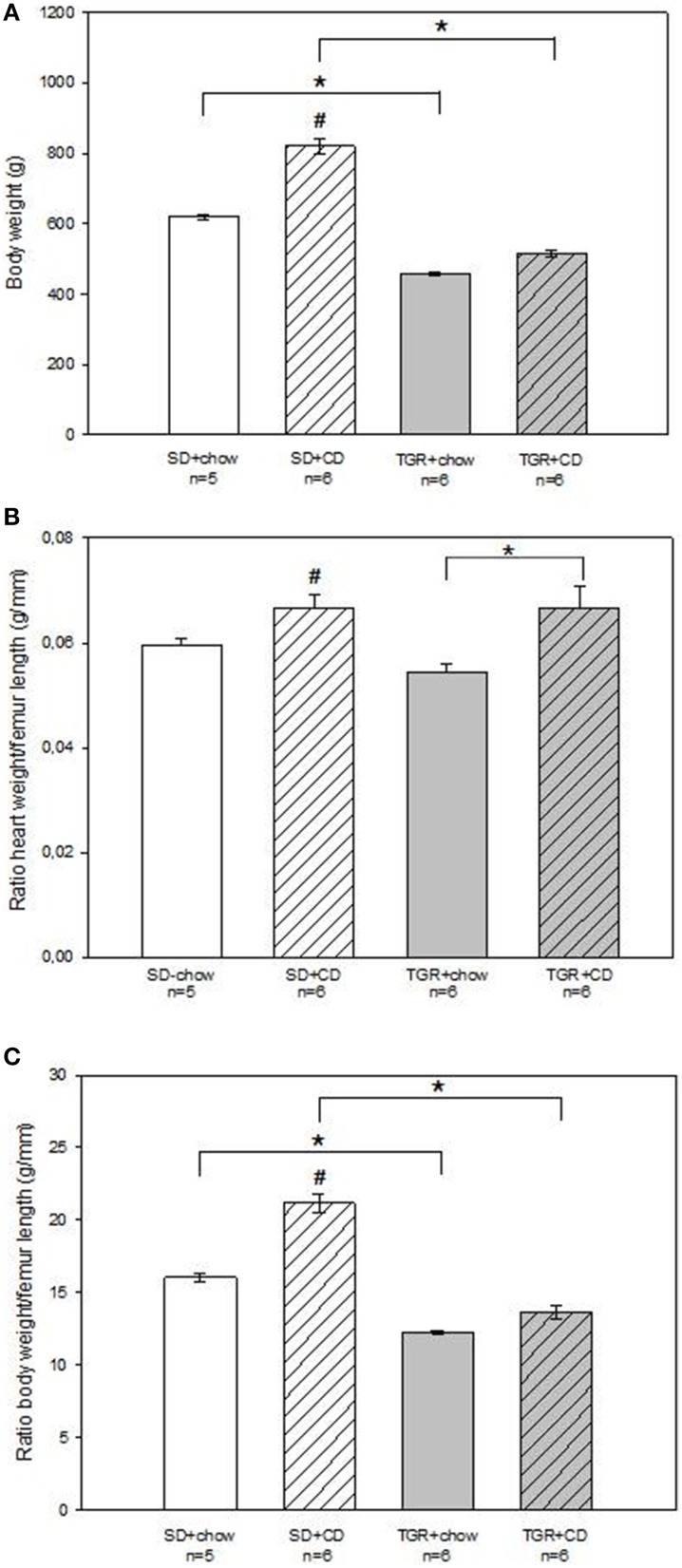
**Body parameters of male wild type Sprague Dawley rats (SD) and transgenic rats (TGR), overexpressing Ang(1-7) after a 5 month feeding period with either standard chow alone or simultaneously chow and cafeteria diet (CD)**. CD increased body weight **(A)**, altered heart weight to femur length ratio **(B)** and elevated body weight to femur length ratio **(C)** in wild type SD rats. In transgenic rats an overexpression of Ang(1-7) prevented the CD-induced effects on body parameters. Data expressed as means ± SEM of *n* experiments. Significant changes vs. chow rats are indicated by a hash key (*p* < 0.05), significant changes between groups are indicated by an asterisk (*p* < 0.05). Body weight was analyzed by Kruskal-Wallis followed by pairwise comparison using the Dwass-Steele-Chritchlow-Fligner. Heart weight to femur length ratio and body weight to femur length ratio were analyzed by ANOVA followed by pairwise comparison using Tukey HSD.

After a 5 month feeding period the body weight, heart weight and femur length of rats were evaluated (Figure 1). The body weight of WT rats, which received chow and CD was significantly elevated compared to WT rats, only fed with chow (Figure 1A). The body weight of TGR+chow rats was significantly lower than that of SD+chow rats. After 5 month of CD feeding TGR rats showed significantly reduced body weight in comparison to the SD+CD group, exhibiting nearly the same body weight like TGR+chow rats. Furthermore, CD feeding of WT rats led to a significant increase in heart weight to femur length ratio compared to SD+chow rats (Figure 1B). In contrast, overexpression of Ang(1-7) inhibited the CD-mediated increase of heart weight to femur length ratio. In addition, CD of WT rats resulted in a significant increase in body weight to femur length ratio compared to SD+chow rats (Figure 1C). Transgenic rats, regardless of feeding, showed significantly decreased body weight to femur length ratio in comparison to SD+chow and SD+CD rats, respectively.

An overexpression of Ang(1-7) clearly prevents from an increase in body weight after CD.

### Hemodynamic measurements

**Figure 2:**
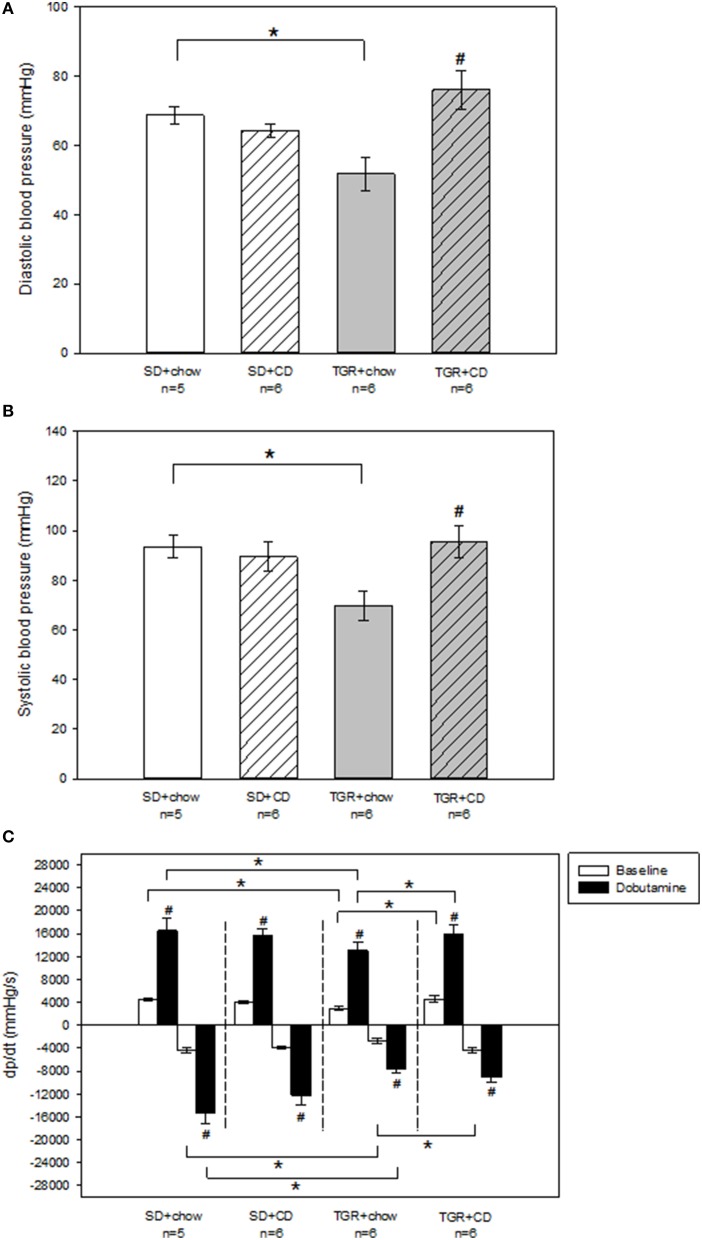
**Hemodynamic measurements of the left ventricle of male wild type Sprague Dawley rats (SD) and transgenic rats (TGR), overexpressing Ang(1-7) after a 5 month feeding period with either standard chow alone or simultaneously chow and cafeteria diet (CD)**. **(A,B)** Recordings of systolic and diastolic blood pressure were carried out under baseline conditions, using a Millar catheter, which was inserted into the left carotid artery. TGR+chow rats showed a significantly impaired systolic and diastolic blood pressure in comparison to SD+chow rats. TGR+CD rats exhibited a significant increase in systolic and diastolic blood pressure compared to TGR+chow rats. Significant changes vs. chow rats are indicated by a hash key (*p* < 0.05), significant changes between groups are indicated by an asterisk (*p* < 0.05). **(C)** Evaluation of dp/dt has been carried out under baseline conditions and under venous injection of dobutamine, using a Millar catheter, which was inserted into the left ventricle via the left carotid artery. CD had no effect on dp/dt(max) and dp/dt(min) in wild type SD rats. TGR+chow rats showed an impairment of dp/dt(max) and dp/dt(min) under baseline conditions and after dobutamine stimulation compared to SD+chow rats. CD in TGR rats resulted in an increase in dp/dt(max) under baseline and after dobutamine stimulation as well as baseline dp/dt(min) compared to TGR+chow rats, whereas dobutamine response regarding dp/dt(min) was unaltered. Significant changes vs. dobutamine stimulation are indicated by a hash key (*p* < 0.05), significant changes between groups are indicated by an asterisk (*p* < 0.05). Data are expressed as means ± SEM of *n* experiments. Hemodynamic parameters were analyzed by Kruskal-Wallis followed by pairwise comparison using the Dwass-Steele-Chritchlow-Fligner.

Echocardiographic measurements revealed that there was no difference in ejection fraction and fractional shortening of the left ventricle between the four groups (Table S1). Moreover, no change of systolic and diastolic blood pressure has been observed after CD feeding in WT rats (Figures 2A,B). Overexpression of Ang(1-7) alone led to a significantly reduced systolic and diastolic blood pressure compared to SD+chow rats. In contrast, overexpression of Ang(1-7) in combination with CD caused significantly elevated systolic and diastolic blood pressures compared to transgenic rats, receiving only chow (Figures 2A,B).

To further analyse left ventricular performance pressure-volume-relationship was investigated. Thereby, pressure-volume-loops were recorded under baseline conditions as well as after stimulation with the beta-adrenoceptor agonist dobutamine to assess beta-adrenergic responsiveness of rat hearts. There was no difference in heart rate under baseline conditions as well as after dobutamine stimulation between the several rat groups (Table S1). Moreover, in WT rats there was no change in endsystolic and enddiastolic left ventricular pressure after CD (Table S1). TGR rats, only fed with chow showed slightly but not significantly reduced baseline endsystolic and enddiastolic left ventricular pressure compared to SD+chow rats. Additionally, dobutamine response regarding endsystolic and enddiastolic left ventricular pressure was also decreased (non-significant) in TGR+chow rats in comparison to SD+chow rats. However, CD feeding of TGR rats resulted in significantly elevated endsystolic and enddiastolic left ventricular pressure under baseline conditions compared to TGR+chow rats. Furthermore, the dobutamine effect regarding enddiastolic left ventricular pressure was also increased in the TGR+CD group in comparison to TGR+chow, whereas endsystolic left ventricular pressure after dobutamine stimulation was not different between these two groups. TGR+CD rats also exhibited a significantly elevated enddiastolic left ventricular pressure under baseline conditions and after dobutamine stimulation compared to SD+CD rats.

No changes in maximal systolic left ventricular contraction velocity (dp/dt(max)) and maximal diastolic left ventricular relaxation velocity (dp/dt(min)) has been observed after CD in WT rats (Figure 2C). In contrast, TGR+chow rats exhibited a significantly impaired baseline dp/dt(max) and dp/dt(min) compared to SD+chow rats (Figure 2C). The dobutamine effect regarding dp/dt(max) and dp/dt(min) was also significantly reduced in the TGR+chow group compared to SD+chow rats. Again, CD led to enhanced dp/dt(max) and dp/dt(min) values under baseline conditions in TGR rats in comparison to TGR+chow animals, whereas the dobutamine response regarding dp/dt(min) was not different between TGR+chow and TGR+CD rats.

Taken together, in contrast to what was expected, hemodynamic measurements revealed that TGR+chow rats showed impairment in left ventricular function in comparison to SD+chow. In addition, CD caused no impairment in cardiac contractility in both, WT and transgenic rats. However, there was an increase in systolic and diastolic blood pressure by CD, but only in transgenic rats as compared to TGR-chow, but not significantly exceeding SD-rats (Figures 2A,B).

### Epicardial mapping

**Figure 3:**
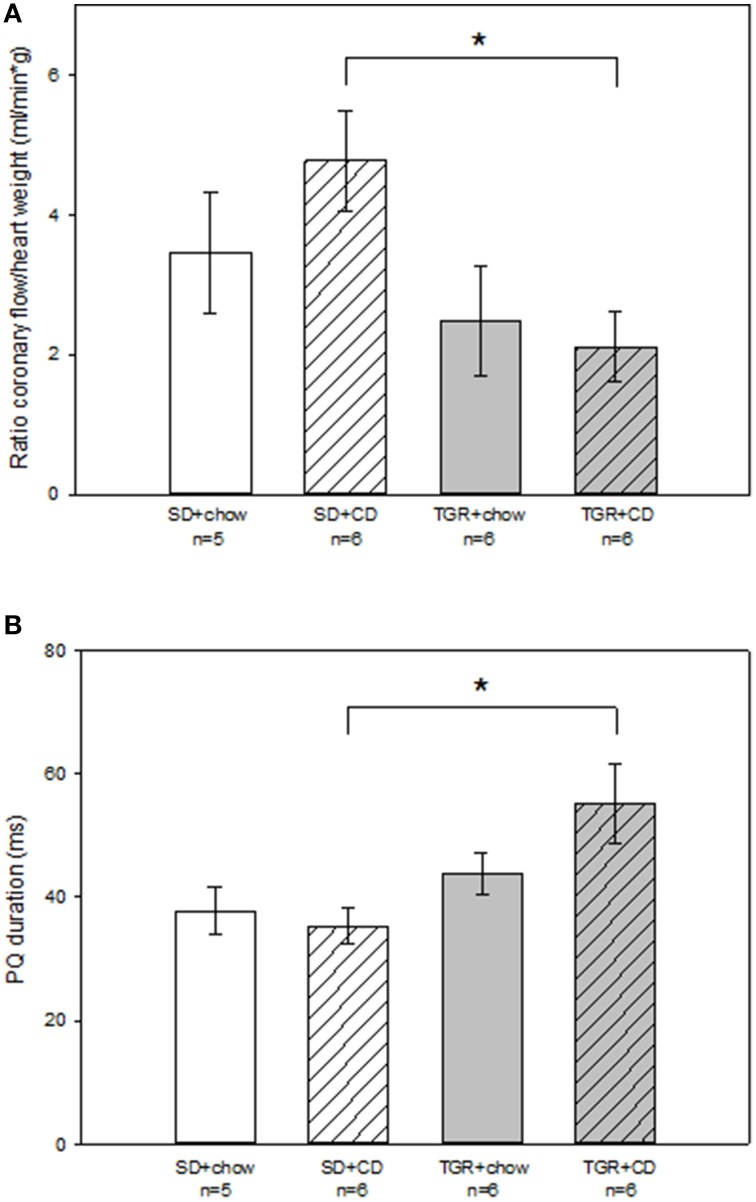
**Epicardial mapping analysis of isolated-perfused hearts of male wild type Sprague Dawley rats (SD) and transgenic rats (TGR), overexpressing Ang(1-7) after a 5 month feeding period with either standard chow alone or simultaneously chow and cafeteria diet (CD)**. Isolated-perfused rat hearts were prepared according to Langendorff-technique. Epicardial mapping was performed as previously described by Dhein et al. (1993). **(A)** Coronary flow was related to heart weight and is given as coronary flow to heart weight ratio. TGR+CD rats showed significantly reduced coronary flow to heart weight ratio compared to SD+CD rats. **(B)** Furthermore, PQ duration of rat hearts was measured. CD in combination with an overexpression of Ang(1-7) led to a significant prolongation of PQ duration in comparison to SD+CD rats. Data expressed as means ± SEM of *n* experiments. Significant changes between groups are indicated by an asterisk (*p* < 0.05). Coronary flow to heart weight ratio and PQ duration were analyzed by Kruskal-Wallis followed by pairwise comparison using the Dwass-Steele-Chritchlow-Fligner.

To investigate the electrical activity of the heart surface epicardial mapping analysis of the isolated-perfused rat hearts were performed (Figure 3; Table S2). Tendentially but not significantly, there was a rise in coronary flow to heart weight ratio in WT rats after CD compared to WT rats, which were fed with chow (Figure 3A). Although, overexpression of Ang(1-7) alone caused a minor decrease in coronary flow to heart weight ratio compared to SD+chow rats, this difference was not statistically significant. Nevertheless, an overproduction of Ang(1-7) after CD significantly abolished the CD-induced increase in coronary flow to heart weight ratio. Results of the 256 recorded epicardial electrocardiograms illustrated that there was no alteration in PQ duration of WT rats, fed with CD. PQ duration of TGR+chow rats remained also unchanged in comparison to SD+chow rats. On the contrary, TGR+CD rats showed a significantly prolonged PQ duration than SD+CD rats, indicating a delayed atrioventricular conduction time (Figure 3B).

Other evaluated parameters such as left ventricular pressure, QRS duration, basic cycle length, total activation time and peak to peak amplitude remained unchanged in all four rat groups (Table S2).

### Histological analysis

**Figure 4:**
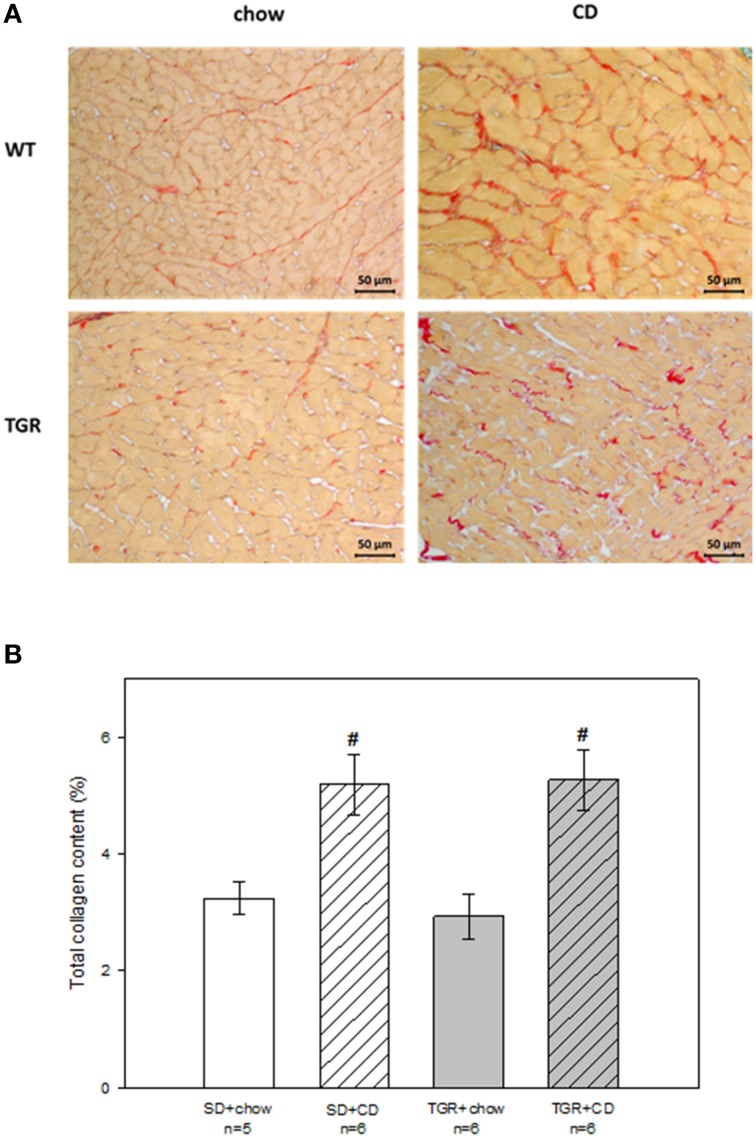
**Total collagen content in the myocardium of male wild type Sprague Dawley rats (SD) and transgenic rats (TGR), overexpressing Ang(1-7) after a 5 month feeding period with either standard chow alone or simultaneously chow and cafeteria diet (CD)**. **(A)** Original histological images of 2 μm sections of rat myocardium stained with Picrosirius Red. **(B)** Quantitative analysis of total collagen content. CD caused myocardial fibrosis in both WT and transgenic rats, without differences between these groups. Data are expressed as means ± SEM of *n* experiments. Significant changes vs. chow rats are indicated by a hash key (*p* < 0.05). Total collagen content was analyzed by ANOVA followed by pairwise comparison using Tukey HSD.

**Figure 5:**
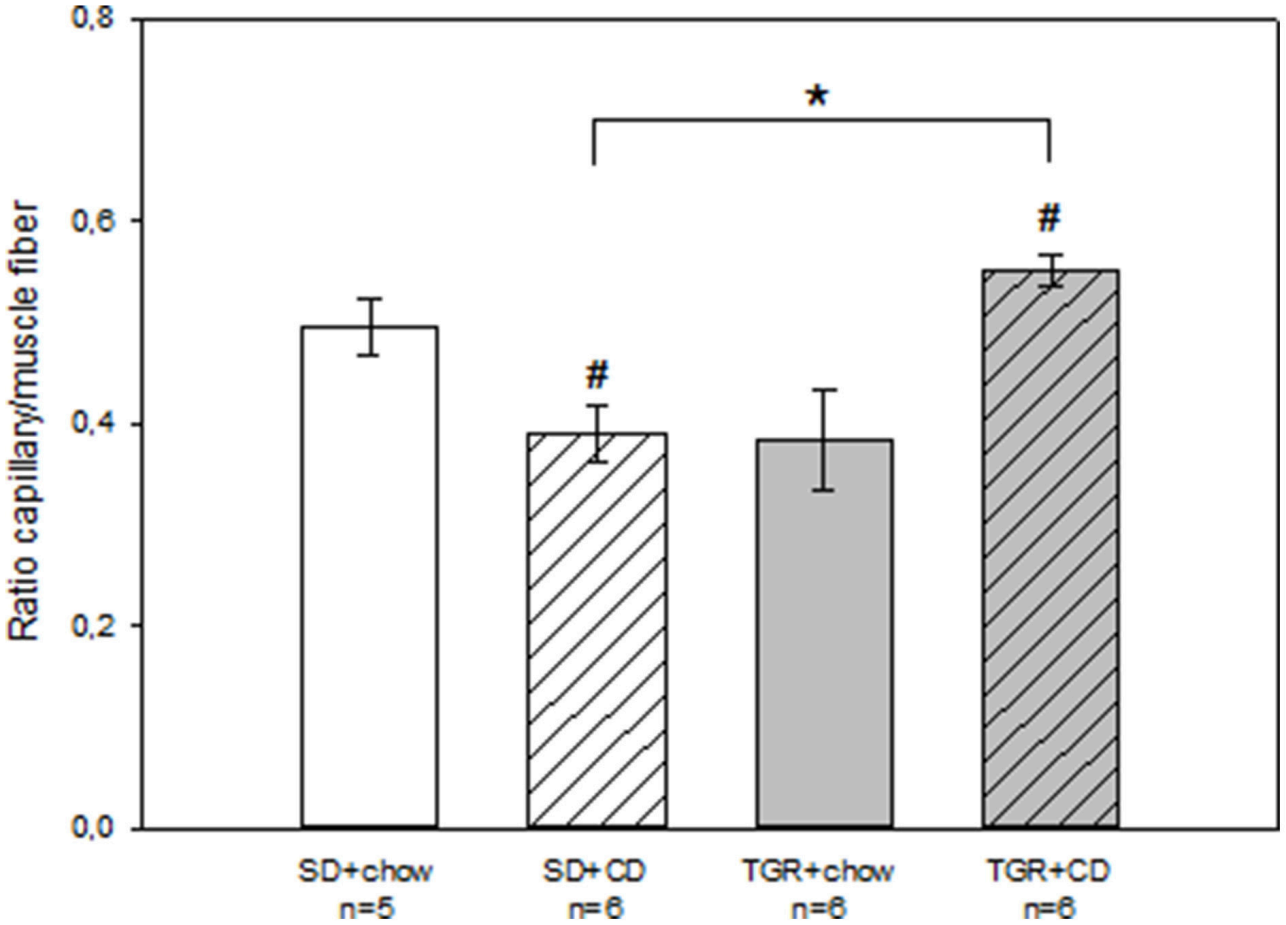
**Capillary to muscle fiber ratio in the myocardium of male wild type Sprague Dawley rats (SD) and transgenic rats (TGR), overexpressing Ang(1-7) after a 5 month feeding period with either standard chow alone or simultaneously chow and cafeteria diet (CD)**. CD led to a decrease in capillary to muscle fiber ratio in WT rats, whereas CD in transgenic rats resulted in an increase in capillary to muscle fiber ratio. Data are expressed as means±SEM of *n* experiments. Significant changes vs. chow rats are indicated by a hash key (*p* < 0.05), significant changes between groups are indicated by an asterisk (*p* < 0.05). Capillary to muscle fiber ration was analyzed by ANOVA followed by pairwise comparison using Tukey HSD.

Histological analysis revealed that rats, fed with CD for 5 months, showed obvious fibrosis in the myocardium, although there was no difference between WT and transgenic rats (Figure 4). Overexpression of Ang(1-7) alone had no effect on myocardial fibrosis. Furthermore, under CD feeding a significant decrease of capillary to muscle fiber ratio could be observed in WT rats (Figure 5). TGR+chow rats exhibited also a reduced capillary to muscle fiber ratio as compared to SD+chow rats (non-significant). In contrast, CD feeding of rats, overexpressing Ang(1-7), resulted in a significant increase in capillary to muscle fiber ratio compared to TGR+chow rats as well as in comparison to SD+CD rats.

In summary, CD caused fibrosis, which could not be prevented by an overproduction of Ang(1-7). Furthermore, CD led to a decrease in capillary to muscle fiber ratio, whereas an overexpression of Ang(1-7) could inhibit this CD-mediated effect and even led to an increase in capillary to muscle fiber ratio.

## Discussion

Obesity is a major global health problem, which becomes more and more important, especially because it is a main risk factor for the development of several cardiometabolic disorders. Thus, the finding, that Ang(1-7) could antagonize diet-induced obesity (Schuchard et al., 2015) was highly interesting, and needed an evaluation whether this effect could be extended to cardiovascular disorders.

The heart is one of the main targets for the novel members of RAAS, the ACE2/Ang(1-7)/Mas receptor axis, but there are controversial results regarding their functional cardiac role. The major findings of our current work are as follows.

1. CD in form of a high sugar diet caused elevated body weight of rats, which was prevented by an overexpression of Ang(1-7). In a previous study Schuchard et al. investigated the same animals, which we inspected in our study. They focused on the effect of CD and overexpressed Ang(1-7) on metabolic parameters of these rats. They could show that the antiobese effect of overexpressed Ang(1-7) was associated with a reduced energy intake but an increased energy expenditure (Schuchard et al., 2015). Furthermore, they observed leptin and insulin resistance, and obesity in SD rats, fed with CD, but not in TGR rats, also received CD (Schuchard et al., 2015).
2. CD had no effect on cardiac performance. However, an overexpression of Ang(1-7) alone resulted in an impairment of hemodynamic parameters, whereas Ang(1-7) overexpression in combination with CD had no obvious effect on cardiac function.
3. CD induced moderate increase in coronary flow to heart weight ratio, whereas transgenic rats, fed with CD, showed a decrease in coronary flow to heart weight ratio.
4. Prolongation of the PQ duration, which represents atrioventricular conduction time, could be observed in rats, overexpressing Ang(1-7) and receiving CD.
5. CD caused myocardial fibrosis, regardless of the genetic background of the rats.
6. CD led to a significantly reduced capillary to muscle fiber ratio. Interestingly, this CD-mediated decrease could be prevented by an overexpression of Ang(1-7).

In conclusion, there was no deteriorating effect of a 5 months CD feeding period on heart function compared to standard chow diet. Furthermore, in this model an overexpression of Ang(1-7) had no beneficial effect on heart function in both, chow and CD fed rats. On the contrary, overexpression of Ang(1-7) alone resulted in an impairment of cardiac performance. However, body weight, coronary flow to heart weight ratio and capillary to muscle fiber ratio were affected by CD and this CD-mediated effect regarding these parameters could be inhibited by Ang(1-7).

First, the lack of cardiovascular effect of CD needs discussion: Others have described the development of hypertension following CD (e.g., Coatmellec-Taglioni et al., 2000; Muntzel et al., 2012; Steiner et al., 2013) and attributed this to increased sympathetic activity (Muntzel et al., 2012) or to the RAAS system (Miesel et al., 2010; Müller-Fielitz et al., 2014). Regarding the cafeteria diet, the authors used 2–20 weeks protocols, so that our protocol seems comparable. However, in contrast to the other authors, we assessed blood pressure in general anesthesia via a Millar catheter, while others used the cuff and collar method. It might be, that due to the hypotensive effects of narcotics our data did not reveal hypertension. Another explanation could be, that the composition of the diet might vary from group to group. Maybe that a longer time of CD-in the composition used by us-is needed to allow the cardiovascular effects to develop. Some of the authors (Miesel et al., 2010) used spontaneously hypertensive rats, which also may exhibit some differences in the blood pressure response. However, our data clearly show that there are only very moderate insignificant changes in the hearts performance, the isolated hearts characteristic, while there is a profibrotic effect in cardiac histology, which however, does not seem to be transduced to the functional level. Thus, from our data, we assume that the CD model is an useful obesity model, but does only partially represent metabolic syndrome as the cardiovascular effects are very small.

In accordance with the results of our work, several studies also reported deleterious effects of the ACE2/Ang(1-7)/Mas receptor axis on the heart. Thus, Donoghue et al. demonstrated that a cardiac overexpression of ACE2 in transgenic mice led to sudden death due to conduction and rhythm disturbances, associated with ventricular arrhythmias (Donoghue et al., 2003). They further revealed that AV block is a main risk factor for the development of ventricular ectopy and sudden cardiac death in the ACE2 transgenic mice. According to our initial idea, we expected CD-induced cardiac fibrosis leading to slowing of ventricular conduction evident from increased TAT, QRS, and reduced PTP-amplitude. Although cardiac fibrosis was induced by CD (Figure 4B) this did not reach functional relevance., since TAT, QRS, and PTP were unaltered in CD rats (Table S2). In comparison to previous studies on aged rabbits, the degree of fibrosis leading to functional consequences needs to be higher (Gottwald et al., 1997; Dhein and Hammerath, 2001). Moreover, from the literature we assumed an antifibrotic effect of Ang(1-7) overexpression, which-according to Figure 4B-could not be seen in our model. Accordingly, we also did not observe significant effects of Ang(1-7) on ventricular conduction spreading.

Regarding acute effects of Ang(1-7) Neves et al. demonstrated that Ang(1-7) dose-dependently (27–210 nmol L^−1^) diminished coronary flow in isolated-perfused rat hearts, without any changes in inotropy or heart rate (Neves et al., 1997). Kumagai et al. observed similar results in isolated hamster hearts (Kumagai et al., 1990). Furthermore, in their study Nerves et al. showed an Ang(1-7)-induced enhancement of reperfusion arrhythmias in isolated-perfused rat hearts (Neves et al., 1997).

One may suggest that possibly the *in vivo* application of dobutamine might have altered the intracellular calcium balance affecting the isolated heart experiment. However, dobutamine has a short half-life and the pre-dobutamine steady state values were reached already during the *in vivo* experiment.

Several studies exist, revealing a cardioprotective role of ACE2 and Ang(1-7) (Ferreira et al., 2001; Pei et al., 2010; Johnson et al., 2011; Dong et al., 2012; Feng et al., 2012; McCollum et al., 2012). In contrast to the above mentioned proarrhythmic effect of high Ang(1-7) concentrations, Ferreira *et al*. detected reduced cardiac arrhythmias induced by ischemia/reperfusion associated with low dose Ang(1-7) if acutely administered (Ferreira et al., 2001). Similar to these observations, De Mello *et al*. described an acute antiarrhythmic action of Ang(1-7) at low concentrations (10^−8^ mol L^−1^), and a proarrhythmic effect of Ang(1-7) at higher concentrations (10^−7^ mol L^−1^) in ventricular preparations of cardiomyopathic hamsters (De Mello et al., 2007).

This biphasic effect of Ang(1-7) is also confirmed by investigations of Sampaio et al., who showed that Wistar rats exhibited an altered regional blood flow distribution, an increase in cardiac output and a reduced total peripheral resistance after acute infusion of low dose of Ang(1-7) (110 fmol min^−1^ 10 min^−1^ iv), whereas opposite effects could be observed after infusion of high dose of Ang(1-7) (11 pmol min^−1^ 10 min^−1^ iv) (Sampaio et al., 2003). However, this again refers to acute and not to chronic effects.

In their study Ferreira et al. observed no differences in heart rate and blood pressure between transgenic rats, overexpressing Ang(1-7) and control rats (Ferreira et al., 2010). Similar to our findings, there was a decrease in coronary flow in transgenic rats. In contrast to the present study, Ferreira et al. showed an improved cardiac function, indicated by elevated dp/dt and-dp/dt and furthermore, an attenuation of the isoproterenol-induced cardiac fibrosis in transgenic rats. Previous studies also indicated an antifibrotic action of Ang(1-7) *in vitro* and *in vivo* (Santos et al., 2004; Ferreira et al., 2010; Dong et al., 2012; McCollum et al., 2012; Acuña et al., 2014). In contrast to these findings, the present study revealed no antifibrotic effect of Ang(1-7) in a transgenic rat model. Moreover, the CD-induced myocardial fibrosis could not be prevented by an overexpression of Ang(1-7). In their study Ferreira *et al*. used a transgenic rat model, which expressed an Ang(1-7)-producing fusion protein, specifically in the heart, which resulted in a cardiac overexpression of Ang(1-7), whereas the plasma Ang(1-7) concentration remained unchanged (Ferreira et al., 2010). In contrast, our transgenic rat model was generated by an Ang(1-7)-producing fusion protein, expressed in testis, which led to an increase in circulating Ang(1-7) levels (Santos et al., 2004; Schuchard et al., 2015). Thus, the findings of Ferreira et al. seemed to be due to local production of cardiac Ang(1-7), whereas our results were rather caused by systemic alterations. Santos et al. (2004) measured Ang(1-7) in the same model as we used, demonstrating elevated Ang(1-7) plasma concentration, but unaltered tissue levels, which may be a result of the short half-life of Ang(1-7), which is about 10 s (Yamada et al., 1998).

Although, other working groups also used the same transgenic rat model as described in the present study there are varying results regarding cardiac function. These working groups described an antiarrhythmic effect, attenuation of hypertension, and a reduction of isoproterenol-induced myocardial fibrosis and cardiac hypertrophy (Santos et al., 2004; Nadu et al., 2008; Santiago et al., 2010). It should be noted, that Santiago et al. only found beneficial effects of Ang(1-7) in rats after deoxycorticosterone acetate (DOCA) salt-induced hypertension, resulting in cardiac dysfunction and remodeling. Moreover, in normotensive transgenic rats there was no cardioprotective effect of Ang(1-7). Interestingly, Santiago et al. described elevated plasma Ang(1-7) levels in normotensive transgenic rats, whereas Ang(1-7) level in the left ventricle remained unchanged in comparison to normotensive non-transgenic animals. Only after induction of DOCA salt hypertension rats exhibited increased left ventricular Ang(1-7). Moreover, evaluation of AngII, Mas and AT_1_ receptor indicated no alteration in mRNA expression in normotensive transgenic rats compared to normotensive non-transgenic rats. In accordance with these findings, Santos et al. demonstrated that the transgene expression in the TGR(A1-7)3293 line is restricted to the testis, leading to an about 4.5-fold increase in Ang(1-7) levels in testis as well as an elevation of venous and arterial Ang(1-7) plasma concentrations, whereas Ang(1-7) level in atrium and the left ventricle was unaltered (Santos et al., 2004). These observations suggest that Ang(1-7) may only exert cardioprotective effects under pathological situations i.e., in the presence of an impaired cardiac function. A balance between Ang(1-7) and AngII levels seems to prevent any beneficial action of Ang(1-7) under physiological conditions. This assumption is confirmed by a study of Mercure et al., who showed that a chronic overproduction of cardiac Ang(1-7) in mice did not affect heart function under normal hemodynamic conditions (Mercure et al., 2008). Regarding heart function, it is known that Ang(1-7) can mitigate isoprenaline effects (Martins Lima et al., 2013), but has no effect on intracellular calcium handling (Dias-Peixoto et al., 2008; Zhou et al., 2015) in normal cardiomyocytes.

In our rat model we also did not observe cardiac dysfunction after high-sugar diet, which could explain the absence of a protective role of the heptapeptide in the heart.

## Limitation

Commonly, obesity results in the development of cardiometabolic disorders in the aging population. The rats used in these experiments were 3 month old when we started with the cafeteria diet and at an age of 8 month cardiac measurements were performed. Thus, future studies with older rats, receiving a longer diet, are needed to investigate whether longer exposition to CD may lead to functionally relevant cardiac fibrosis. Furthermore, we used a transgenic rat model, which expressed an Ang(1-7)-producing fusion protein in testis, leading to an overexpression of testicular and plasma Ang(1-7). Thus, our findings regarding cardiac performance and structure are ascribed to the action of systemic production of Ang(1-7) rather than a local production of Ang(1-7) in the heart.

## Clinical perspective

Previous studies demonstrated that inhibition of the ACE/AngII/AT_1_ receptor axis attenuate metabolic abnormalities, indicating the ACE/AngII/AT_1_ receptor arm as a therapeutic strategy for the treatment of metabolic diseases (Feltenberger et al., 2013; Santos et al., 2013a,b; Santos and Simoes, 2014). Furthermore, there is evidence suggesting the ACE2/Ang(1-7)/Mas receptor axis as a physiological antagonist of the conventional ACE/AngII/AT_1_ receptor arm (Patel et al., 2014).

Therefore, we wanted to investigate the effect of a high-sugar diet and an overexpression of Ang(1-7) on cardiac performance and remodeling in an experimental rat model of obesity.

Our results indicate that an overexpression of circulating Ang(1-7) protects rats against a CD-induced increase in body weight, but does not affect heart function. An overexpression of Ang(1-7) alone led to an impaired cardiac performance.

Due to its observed antiobese effect Ang(1-7) seems to be a potential therapeutic option in treating obesity. This could be relevant for human obesity, which may cause metabolic as well as cardiovascular diseases in the long term. However, although we did observe clear anti-obesity effects of Ang(1-7) we could not confirm any cardioprotective effect. Even in contrast, the cardiac performance was lower in the transgenics. Thus, from our point of view it is too early to state, that Ang(1-7) may be the pathway of choice to treat cardiovascular aspects of the metabolic syndrome.

## Author contributions

SD, FS, KB, AS, WR, MB, and ID conceived and designed the experiments for this manuscript. SD, FS, KB, AS performed the experiments, analyzed and interpreted the data of this work. Transgenic rats were generated by MB. We received all investigated rats from WR.

### Conflict of interest statement

The authors declare that the research was conducted in the absence of any commercial or financial relationships that could be construed as a potential conflict of interest.
