# Supplementary material for: Effect of Angiotensin(1-7) on Heart Function in an Experimental Rat Model of Obesity
Source: Front Physiol. 2015 Dec 21;6:392. doi: 10.3389/fphys.2015.00392 (PMC4685089; doi:10.3389/fphys.2015.00392)
Supplement: Supplementary file 1 [file Table1.DOCX]

Supplementary Material

Effect of angiotensin(1-7) on heart function in an experimental rat model of obesity

**Katja Blanke^1^, Franziska Schlegel^2^, Walter Raasch^3^, Michael Bader^4^, Ingo Dähnert^1^, Stefan Dhein^2^, Aida Salameh^1*^**

*** Correspondence:** Prof.Dr. Aida Salameh, Heart Center Leipzig, Department of Pediatric Cardiology, Strümpellstraße 39, 04289 Leipzig, Germany, aida.salameh@medizin.uni-leipzig.de

# Supplementary Figures and Tables

## Supplementary Tables

Supplementary Table 1. Heart weight and echocardiography of the left ventricle of male wild type Sprague Dawley rats (SD) and transgenic rats (TGR), overexpressing Ang(1-7) after a five month feeding period with either standard chow alone or simultaneously chow and cafeteria diet (CD). For echocardiographic analysis the left ventricle was monitored in the parasternal short axis view in the M- mode. Arterial blood pressure was measured using a Millar catheter, inserted into the left carotid artery. Endsystolic and enddiastolic left ventricular pressure was recorded under baseline conditions and after dobutamine stimulation, using the Millar catheter, which was fit into the left ventricle. After rats were finalized, hearts were removed and heart weights were determined.

Data expressed as means±SEM of n experiments. Significant changes vs. TGR+CD rats are indicated by a hash key (p<0.05), significant changes by the application of dobutamine are indicated by an asterisk (p<0.05). Ejection fraction and fractional shortening as well as endsystolic and enddiastolic left ventricular pressure were analyzed by Kruskal- Wallis followed by pairwise comparison using the Dwass- Steele- Chritchlow- Fligner. Heart weight, femur length and heart rate were analyzed by ANOVA followed by pairwise comparison using Tukey HSD.

HW, heart weight; FL, femur length; EF, ejection fraction; FS, fractional shortening; HR, heart rate; LVESP, endsystolic left ventricular pressure; LVEDP, enddiastolic left ventricular pressure.

|  | **SD+chow**  **(n=5)** | **SD+CD**  **(n=6)** | **TGR+chow**  **(n=6)** | **TGR+CD**  **(n=6)** |
| --- | --- | --- | --- | --- |
| **HW [g]** | 2.3±0.10 | 2.6±0.09 | 2.0±0.07# | 2.5±0.14 |
| **FL [mm]** | 39±0.49 | 39±0.54 | 37±0.15 | 38±0.87 |
| **EF [%]** | 70±2.02 | 71±0.82 | 69±1.02 | 68±0.87 |
| **FS [%]** | 35±1.46 | 36±0.65 | 34±0.73 | 34±0.66 |
| **HR [bpm]**  baseline | 216±6.36 | 220±5.34 | 231±5.97 | 223±5.60 |
| dobutamine | 286±9.72🞹 | 287±5.32🞹 | 346±29.89🞹 | 298±15.19🞹 |
| **LVESP [mmHg]**  baseline | 79±4.75 | 78±4.26 | 64±6.53# | 95±10.21 |
| dobutamine | 146±14.22🞹 | 146±16.01🞹 | 121±11.04🞹 | 155±12.69🞹 |
| **LVEDP [mmHg]**  baseline | 8±1.54 | 6±0.92# | 5±0.81# | 9±0.86 |
| dobutamine | 7±1.22 | 6±0.44# | 5±0.86# | 10±0.92 |
