# Supplementary material for: Effect of Angiotensin(1-7) on Heart Function in an Experimental Rat Model of Obesity
Source: Front Physiol. 2015 Dec 21;6:392. doi: 10.3389/fphys.2015.00392 (PMC4685089; doi:10.3389/fphys.2015.00392)
Supplement: Supplementary file 2 [file Table2.DOCX]

Supplementary Material

Effect of angiotensin(1-7) on heart function in an experimental rat model of obesity

**Katja Blanke^1^, Franziska Schlegel^2^, Walter Raasch^3^, Michael Bader^4^, Ingo Dähnert^1^, Stefan Dhein^2^, Aida Salameh^1*^**

*** Correspondence:** Prof.Dr. Aida Salameh, Heart Center Leipzig, Department of Pediatric Cardiology, Strümpellstraße 39, 04289 Leipzig, Germany, aida.salameh@medizin.uni-leipzig.de

# Supplementary Figures and Tables

## Supplementary Tables

**Supplementary Table 2. Epicardial mapping analysis of isolated- perfused hearts of male wild type Sprague Dawley rats (SD) and transgenic rats (TGR), overexpressing Ang(1-7) after a five month feeding period with either standard chow alone or simultaneously chow and cafeteria diet (CD).** Isolated- perfused rat hearts were prepared according to Langendorff- technique. Electrophysiology of these hearts was evaluated by epicardial mapping (Dhein *et al.* 1993). Data expressed as means±SEM of n experiments. Coronary flow, QRS duration and peak to peak amplitude were analyzed by Kruskal- Wallis followed by pairwise comparison using the Dwass- Steele- Chritchlow- Fligner. Developed left ventricular pressure, end-diastolic pressure, basic cycle length, heart rate, and total activation time were analyzed by ANOVA followed by pairwise comparison using Tukey HSD.

CF, coronary flow; LVP, developed eft ventricular pressure; EDP, end-diastolic pressure, BCL, basic cycle length; HR, heart rate; TAT, total activation time; PTP, peak to peak amplitude.

|  | **SD+chow**  **(n=5)** | **SD+CD**  **(n=6)** | **TGR+chow**  **(n=6)** | **TGR+CD**  **(n=6)** |
| --- | --- | --- | --- | --- |
| **CF [ml/min/g]** | 3.54±0.62 | 4.77±0.72# | 2.48±0.79 | 2.10±0.61 |
| **Developed LVP [mmHg]** | 85±5.16 | 83±3.63 | 74±8.39 | 97±9.39 |
| **EDP [mmHg]** | 7.0±0.7 | 7.5±0.9 | 7.8±1.6 | 7.7±0.9 |
| **QRS [ms]** | 14±2.07 | 14±1.95 | 19±1.29 | 18±2.05 |
| **BCL [ms]** | 212±34.99 | 227±36.95 | 229±21.85 | 288±51.11 |
| **HR [bpm]** | 310±34.40 | 292±36.32 | 273±24.60 | 237±38.63 |
| **TAT [ms]** | 9±1.51 | 7±1.27 | 8±0.56 | 9±0.67 |
| **PTP [mV]** | 6±0.37 | 7±0.29 | 7±0.10 | 6±0.30 |
